# Screening for Gestational Diabetes Mellitus: Is There a Need for Early Screening for All Women in Developing Countries?

**DOI:** 10.7759/cureus.35533

**Published:** 2023-02-27

**Authors:** Oluwasegun A Akinyemi, Ofure V Omokhodion, Mojisola E Fasokun, Deborah Makanjuola, Idowu P Ade-Ojo, Adebayo A Adeniyi

**Affiliations:** 1 Health Policy and Management, University of Maryland School of Public Health, College Park, USA; 2 Surgery, Howard University, Washington, DC, USA; 3 Obstetrics and Gynecology, University College Hospital, Ibadan, NGA; 4 Epidemiology and Public Health, University of Alabama at Birmingham, Birmingham, USA; 5 Epidemiology, University of Alabama at Birmingham, Birmingham, USA; 6 Obstetrics and Gynecology, Ekiti State University Teaching Hospital, Ado Ekiti, NGA; 7 Obstetrics and Gynecology, Federal Teaching Hospital Ido-Ekiti, Ido Ekiti, NGA

**Keywords:** oral glucose tolerance test, diabetes mellitus, blood-glucose, prevalence, ogtt, gestational diabetes mellitus, early universal screening

## Abstract

Background: Gestational diabetes mellitus (GDM) is associated with significant adverse pregnancy outcomes. Early diagnosis and treatment have been proven to reduce adverse pregnancy outcomes among women diagnosed with GDM. Current guidelines recommend routine screening for GDM at 24-28 weeks of pregnancy, with early screening offered to those considered high risk. However, risk stratification may not always be helpful for those who would benefit from early screening, especially in non-Western settings.

Aim: To determine the need for early screening for GDM among pregnant women attending antenatal clinics in two tertiary hospitals in Nigeria.

Methods: We conducted a cross-sectional study from December 2016 to May 2017. We identified women who presented at the antenatal clinics of the Federal Teaching Hospital Ido-Ekiti and Ekiti State University Teaching Hospital, Ado Ekiti. A total of 270 women who fulfilled the study inclusion criteria were enrolled. The 75 g oral glucose tolerance test was used to screen participants for GDM before 24 weeks and between 24 and 28 weeks for those who screened negative before 24 weeks. Pearson's chi-square test, Fisher's exact test, independent t-test, and Mann-Whitney U test were utilized in the final analysis.

Results: The median age of the women in the study was 30 (interquartile range: 27-32) years. Of our study participants, 40 (14.8%) were obese, 27 (10%) had a history of diabetes mellitus in a first-degree relative, and three (1.1%) women had a previous history of GDM. Twenty-one women (7.8%) were diagnosed with GDM, and six (28.6%) were diagnosed before 24 weeks. Women diagnosed with GDM before 24 weeks were older (37 years; interquartile range: 34-37) and more likely to be obese (80.0%). A significant number of these women also had identifiable risk factors for GDM: previous GDM (20.0%), family history of diabetes mellitus in a first-degree relative (80.0%), prior delivery of fetal macrosomia (60.0%), and previous history of congenital fetal anomaly (20.0%).

Conclusion: The findings from the present study did not justify universal screening for GDM in all pregnant women. Patients diagnosed before the 24-28 weeks of universal screening are more likely to have significant risk factors for GDM and, therefore, would have been selected for screening based on the risk factor screening.

## Introduction

Gestational diabetes mellitus (GDM) is any glucose intolerance with onset or first recognition in pregnancy [1]. It is one of the most common pregnancy complications and affects about 1-14% of pregnancies worldwide [2]. It is associated with maternal and fetal complications, including fetal macrosomia, stillbirth, birth trauma, preeclampsia, cesarean delivery, post-operative infections, neonatal hypoglycemia, and an increased risk of developing type 2 diabetes mellitus [3-5]. The risk factors for GDM include previous history of GDM, family history of diabetes, and race. Selective screening is carried out in some European countries due to lower costs [1], and the effectiveness of early screening based on maternal risk factors has been supported by the literature [6]. However, screening in the USA is more universal and is associated with lower costs in the long term [7].

Screening for GDM is usually carried out between 24 and 28 weeks for all pregnant women with a 75 g oral glucose tolerance test (OGTT), as proposed by the International Association of Diabetes and Pregnancy Study Groups (IADPSG), and this was adopted by the World Health Organization (WHO) in 2013. Risk-based screening is practiced in Nigeria, where the prevalence of GDM is high [8], and is targeted toward women with an increased risk of GDM at 24-28 weeks [9]. Early screening is usually reserved for women with a high risk of GDM, and it has been found that the prevalence of GDM in the first and second trimesters is significant and that up to one in 20 cases of GDM may be missed by risk-based screening alone [9].

Although recent studies have reported the need for earlier screening, there is not enough evidence to support the revision of current guidelines, especially among low-risk pregnant women [10,11]. Early detection and treatment improve pregnancy outcomes, making universal early screening beneficial. This study, therefore, aims to determine if early universal screening of GDM is beneficial, by identifying the prevalence of GDM diagnosis before 24 weeks. It will also determine if the diagnosis of GDM correlates with identifiable risk factors.

## Materials and methods

The study was conducted among pregnant women attending antenatal clinics at the Ekiti State University Teaching Hospital (EKSUTH), Ado Ekiti, and the Federal Teaching Hospital (FTH) Ido-Ekiti, Ekiti State, Western Nigeria. These women attended antenatal care (ANC) between December 2016 and May 2017. Women were excluded if they had no reliable way to date the pregnancy. Women who declined to consent to the study were also excluded. Written informed consent was sought from the study participants. A total of 280 women participated in the study, with 10 lost to follow-up. The 75 g OGTT was performed according to the WHO recommendation on the appointed date for the test. Socio-demographic information was extracted from the case notes of the patients. GDM was diagnosed using the IADPSG criteria. Patients with at least one of the following were classified as having GDM: fasting plasma glucose ≥ 92 mg/dl (≥5.2 mmol/l), one-hour plasma glucose ≥ 180 mg/dl (≥10 mmol/l), or two-hour plasma glucose ≥ 153 mg/dl (≥8.5 mmol/l). The study outcome variable was a diagnosis of GDM based on the IADPSG criteria. This was done twice in pregnancy, before the 24th week, and women who were negative during the first screening had a repeat screening and diagnosis between 24 and 28 weeks. The independent variables included the body mass index (BMI), calculated from measured pre-pregnancy weight (kg) and height (m) at the first visit. Other variables include patients' age, education status, race/ethnicity, and risk factors. We received permission to conduct this study from both the EKSUTH, Ado Ekiti and FTH Ido-Ekiti Institution Review Boards (EKSUTH/A67/2016/01/004/ERC/2016/01/12/02A).

Statistical analysis

We utilized Pearson's chi-square test to evaluate the relationship between the studied variables and the occurrence of GDM before 24 weeks and between 24 and 28 weeks. A two-tailed p-value < 0.05 was considered statistically significant. All statistical analyses were performed using STATA version 16 (StataCorp LLC, College Station, TX).

## Results

Table 1 shows the study participants' demographic characteristics and risk factors. Among the 270 women who participated in the study, 42 (15.6%) were >35 years old at the time of the survey. The prevalence of GDM in the sample population was 7.8%, with 21 positive cases. Most of the study participants had normal pre-pregnancy BMI (184, 68.1%), 55 (20.4%) were overweight, 18 (6.7%) were obese, and only 13 (4.8%) were underweight. Fetal macrosomia was relatively common, with 32 (11.9%) women having a previous delivery of a child with a delivery weight > 4 kg. Only 27 (10%) women had a family history of diabetes in a first-degree relative. There were six (2.2%) cases of chronic hypertension, 10 (3.7%) previous records of intrauterine fetal death or stillbirths, and four (1.5%) prior histories of delivery of a baby with a congenital abnormality. Only three (1.1%) women had a previous history of GDM.

**Table 1:** Determinants of gestational diabetes mellitus (24-28 weeks diagnosis) GDM, gestational diabetes mellitus; p < 0.05, statistical significance.

Variables	Total population	GDM (24-28 weeks)	Normal	P-value
	(n = 270)	(n = 21)	(n = 249)	
Median age (interquartile range)	30 ( 27-32)	37 (36-38)	30 (27-32)	<0.001
Age				<0.001
≤35 years	228 (84.4%)	4 (19.1%)	224 (90.0%)	
>35 years	42 (15.6%)	17 (80.9%)	25 (10.0%)	
Median pre-pregnancy body mass index (interquartile range)	22.8 (20.8-25.3)	29.1 (24.8-31.1)	22.5 (20.3-24.9)	<0.001
Body mass index (kg/m^2^)				<0.001
Normal body mass index	139 (51.5%)	1 (4.8%)	138 (55.4%)	
Overweight	91 (33.7%)	6 (28.6%)	85 (34.1%)	
Obese	40 (14.8%)	14 (66.7%)	26 (10.4%)	
Previous gestational diabetes mellitus	3 (1.1%)	3 (14.3%)	0 (0.0%)	<0.001
Family history of diabetes mellitus	27 (10.0%)	17 (81.0%)	10 (4.0%)	<0.001
Chronic hypertension	6 (2.2%)	2 (9.5%)	4 (1.6%)	0.02
Macrosomia	32 (11.9%)	13 (61.9%)	19 (7.6%)	<0.001
Stillbirth	6 (2.2%)	0 (0.0%)	6 (2.4%)	0.47
Spontaneous miscarriage	37 (13.7%)	3 (14.3%)	34 (13.7%)	0.94
Previous congenital anomaly	4 (1.5%)	2 (9.5%)	2 (0.8%)	<0.001
Intrauterine fetal death	4 (1.5%)	2 (9.5%)	2 (0.8%)	<0.001

Table 2 describes the association between some selected traditional risk factors of GDM and the diagnosis of the disease before 24 weeks. Women diagnosed before 24 weeks were older, with a median age of 37 (interquartile range: 34-37) years, 80.0% of these women were obese, and a significant proportion of the women have traditional risk factors for GDM, including previous GDM (20.0%), family history of diabetes (80.0%), prior delivery of a baby weighing ≥ 4 kg (60.0%), and previous history of congenital fetal anomaly (20.0%).

**Table 2:** Determinants of gestational diabetes mellitus (<24 weeks diagnosis) GDM, gestational diabetes mellitus; p < 0.05, statistical significance.

Variable	Total	GDM (<24 weeks)	Normal	P-value
	(n = 270)	(n = 6)	(n = 264)	
Median age (interquartile range)	30 (27-32)	37 (34-37)	30 (27-32)	<0.001
Age				<0.001
≤35 years	228 (84.4%)	2(40.0%)	226 (85.3%)	
>35 years	42 (15.6%)	3 (60.0%)	39 (14.7%)	
Median pre-pregnancy body mass index (interquartile range)	22.8 (20.8-25.3)	29.3 (29.1-31.3)	22.8 (20.8-25.0)	<0.001
Obese	40 (14.8%)	4 (80.0%)	36 (13.6%)	<0.001
Previous gestational diabetes mellitus	3 (1.1%)	1 (20.0%)	2 (0.8%)	<0.001
Family history of diabetes mellitus	27 (10.0%)	4 (80.0%)	23 (8.7%)	<0.001
Chronic hypertension	6 (2.2%)	0 (0.0%)	6 (2.3%)	0.73
Macrosomia	32 (11.9%)	3 (60.0%)	29 (10.9%)	<0.001
Spontaneous miscarriage	37 (13.7%)	1 (20.0%)	36 (13.6%)	0.7
Stillbirth	6 (2.2%)	0 (0.0%)	6 (0.73)	0.73
Intrauterine fetal death	4 (1.5%)	0 (0.0%)	4 (1.5%)	0.78
Previous congenital anomaly	4 (1.5%)	1 (20.0%)	3 (1.1%)	<0.001

## Discussion

In the present study, the prevalence of GDM was 7.8%, which is lower than the pooled prevalence of GDM in Nigeria reported in a systematic review (11%), and this was attributed to differences in female characteristics of the study participants and differences in the screening methods utilized [8]. A prevalence of 5.2% was also reported and it was concluded that the prevalence of GDM in Nigeria was on the rise and that the disparities in prevalence reports could be attributed to better screening tools, better screening policies, and increased exposure to risk factors [9].

About one in every three women diagnosed with GDM was diagnosed before 24 weeks, which is lower than previous findings that one in every two women diagnosed with GDM was diagnosed in the first and second trimesters [9]. These findings support early screening for GDM, which will detect women who develop GDM before 24 weeks and would have been missed by universal screening after 24 weeks. However, the United States Preventive Services Task Force (USPSTF) stated that there was insufficient evidence to assess the balance of benefits and harms of screening for gestational diabetes in asymptomatic pregnant persons before 24 weeks of gestation and recommended screening for gestational diabetes in asymptomatic pregnant persons at 24 weeks of gestation or after [12].

In the present study, there was a significant association between risk factors and diagnosis of GDM before the 24th week of pregnancy. The women diagnosed with GDM before 24 weeks were generally older and had a higher pre-pregnancy body mass index. A higher proportion of these women were obese and had traditional risk factors for GDM, similar to the findings of a case-control study carried out in Iran, where the prevalence of GDM was higher than in most regions in the world [13]. The authors found that a family history of type 2 diabetes mellitus was the most important risk factor for GDM, with advanced maternal age and obesity also having significant associations with a diagnosis of GDM. In our study, a previous history of fetal macrosomia was the commonest risk factor for GDM. These findings align with the literature, which supports the association between risk factors and the diagnosis of GDM irrespective of gestational age [6,14] and justifies risk stratification as a reliable way to decide whom to offer early screening. This further implies that older women who are obese or overweight should be the target of early screening efforts and lifestyle modifications. Our findings of a lack of association between some risk factors for GDM, such as spontaneous miscarriage and stillbirth, and diagnosis before 24 weeks may be due to smaller numbers and the multifactorial etiology of these conditions.

A retrospective study done in Estonia, where risk-based screening is practiced, showed that women with risk factors for GDM who screened negative with OGTT were still at risk of excessive weight gain and large for gestational age (LGA) deliveries, which are in turn associated with adverse pregnancy outcomes [15]. This may be explained by the confounding effect of other risk factors such as obesity on LGA and excessive weight gain and implies that women with risk factors for GDM who screen negative should not be dismissed and should still be provided with management options for reducing gestational weight gain. It is of interest that some of the women diagnosed with GDM did not have identifiable risk factors, and this is also supported by literature findings [16,17]. These studies overall argue for universal screening of all women for GDM regardless of risk factor stratification.

Clearly, both universal screening and risk-based screening have benefits that are applicable in different settings. In settings where the prevalence of GDM is high and resources are available, universal screening would be beneficial in the long term, and in resource-limited settings like Nigeria, risk-based screening may be more appropriate. Irrespective of the setting, however, the adverse pregnancy outcomes associated with GDM can be avoided by initiating behavioral lifestyle modifications during the antenatal period [18] and incorporating these interventions into routine ANC.

A major obstacle in detecting and managing GDM in Nigeria is low ANC attendance. According to the Nigerian Demographic and Health Survey, only 67% of women have at least one ANC throughout pregnancy, 57% have at least four ANC visits, and more than a third do not have formal ANC [19]. There are obvious benefits of ANC in improving pregnancy outcomes in women with GDM. Adverse pregnancy outcomes such as fetal distress, instrumental delivery, cesarean delivery, poor Apgar scores, and neonatal hypoglycemia, which lead to neonatal intensive care unit (NICU) admission, can be avoided by improving maternal glycemic control. Glycemic control can be achieved in the antenatal period by lifestyle modifications such as dietary restriction and glucose monitoring [20,21].

The current guidelines for screening for GDM at 24-28 weeks are based on evidence that the pathogenic mechanism for GDM (insulin resistance) begins at this period [22,23]. However, other studies have shown that insulin resistance in pregnant women may start as early as 14 weeks of gestation [24]. Overall, there is insufficient evidence to demonstrate a clear benefit, especially in low-resource settings, of the need for early screening [25]. Our study highlights the importance of screening women with risk factors for GDM irrespective of their gestational age and alludes to the importance of initiating intervention in women with risk factors for GDM who may screen negative.

It is vital to interpret the findings from this study with caution. First, as a two-center study, the results may not be generalizable to the whole country. Since this is a cross-sectional study, it is challenging to make categorical statements on screening since we cannot establish temporality or causality from cross-sectional studies. Lastly, the authors cannot rule out specific biases such as reporting and selection bias. However, the strengths of the present study include that it addresses a knowledge gap about the association between risk factors of GDM and diagnosis of GDM before 24 weeks. Although our findings do not support early universal screening, it emphasizes the need for focused intervention on all women with risk factors for GDM.

## Conclusions

The prevalence of GDM is on the rise globally and is associated with adverse pregnancy outcomes, which can be prevented through early detection and treatment. Our findings agree with other studies that justify risk-based screening for women with GDM. Women with GDM diagnosed before 24-28 weeks when universal screening occurs are more likely to have significant risk factors for GDM and, therefore, would have been selected for screening based on the risk factor screening algorithm. More evidence supporting the effectiveness of screening for GDM before 24 weeks is necessary if policies are to be modified to accommodate this.
